# Revolutionizing Severe Malaria Management: The Role of CytoSorb® Hemoadsorption in Treating Malaria-Induced Liver Dysfunction

**DOI:** 10.7759/cureus.77696

**Published:** 2025-01-20

**Authors:** Rosanna Carmela De Rosa, Antonio Romanelli, Roberto Giurazza, Fabrizio Falso, Gianfranco Viola

**Affiliations:** 1 Department of Anesthesia and Intensive Care, Azienda Ospedaliera Rilievo Nazionale (AORN) Ospedali dei Colli - "D. Cotugno" Hospital, Naples, ITA; 2 Department of Critical Care, Azienda Ospedaliera Rilievo Nazionale (AORN) Ospedali dei Colli, Naples, ITA; 3 Department of Anesthesia and Critical Care, Azienda Ospedaliera Universitaria (AOU) San Giovanni di Dio e Ruggi d'Aragona, Salerno, ITA; 4 Department of Anesthesia and Critical Care, Azienda Ospedaliera Rilievo Nazionale (AORN) Ospedali dei Colli, Naples, ITA

**Keywords:** case reports, cytosorb®, hemoadsorption, liver dysfunction, malaria

## Abstract

Malaria, caused by *Plasmodium falciparum *(PF), can lead to severe liver dysfunction and hyperbilirubinemia, worsening the prognosis. A 53-year-old male patient with malaria-related liver dysfunction and severe hyperbilirubinemia was treated with extracorporeal hemoadsorption (EHA) with the CytoSorb^®^ filter (CytoSorbents, Monmouth Junction, NJ), marking a turning point in his treatment. This filter, by removing inflammatory mediators and bilirubin, significantly reduced bilirubin levels and improved the patient's clinical condition. This intervention facilitated a bridging therapy, improving symptoms and preventing organ damage during antimalarial treatment. CytoSorb^®^ in EHA shows promise in treating malaria-induced liver dysfunction, suggesting the need for further research on its broader clinical application.

## Introduction

Human malaria is caused by microorganisms of the *Plasmodium* species, specifically *Plasmodium falciparum* (PF), *P. vivax*, *P. ovale*, *P. malariae*, and *P. knowlesi*. It is spread through bites of infected female *Anopheles* mosquitoes. The parasites mature and reproduce in the host liver. Severe malaria by PF results in multiorgan dysfunction, encephalopathy, and shock. In 1970, the World Health Organization (WHO) declared Italy malaria-free, but travelers can import the disease after visiting endemic tropical and subtropical areas with hot-humid conditions [1]. Signs include periodic fever, chills, rigidity, sweating, diarrhea, abdominal pain, respiratory distress, confusion, convulsions, hemolytic anemia, splenomegaly, and kidney and liver dysfunction.

Hyperbilirubinemia, mainly unconjugated, is a common feature of PF malaria and is attributed to the hemolysis of parasitized and non-parasitized erythrocytes and liver cell damage. The sporozoite form of the parasite infects liver cells, causing congestion, sinusoidal blockage, and cellular inflammation [2,3]. In acute liver failure, hydrophobic (unconjugated bilirubin, bile acids, phenols, aromatic amino acids, and fatty acids) and hydrophilic (ammonium and pro-inflammatory cytokines) toxic substances accumulate in the blood, increasing the incidence of complications and worsening the prognosis [4].

The purpose of extracorporeal hemoadsorption (EHA) technologies in case of hyperbilirubinemia and liver dysfunction is to remove toxic substances using adsorbent filters. The pores of the adsorbent filters act as molecular sieves and prevent the entry of molecules larger than the specific cutoff. Adsorbents based on styrene-divinylbenzene polymers with 15 nm pores (1 nm ~0.66 kDa) are suitable for the removal of cytokines, while those with 30-40 nm pores are the best choice for the removal of albumin-related toxins. The CytoSorb® (CytoSorbents, Monmouth Junction, NJ) system effectively removes bilirubin with minimal albumin loss. Indeed, it can break the albumin-bilirubin complex and adsorb bilirubin irreversibly. Case reports have demonstrated the efficacy of CytoSorb® in removing both unconjugated bilirubin (0.6 kDa) and conjugated bilirubin (0.8 kDa), particularly in patients with septic shock and severe hepatic failure [5,6].

Here, we reported a case of severe liver failure caused by PF malaria successfully treated with EHA with CytoSorb® filter. The patient showed significant clinical improvement and was subsequently discharged to home in good condition.

## Case presentation

A 53-year-old man was hospitalized (Cotugno Hospital, Naples, Italy) for fever, diarrhea, dyspnea, and hyperbilirubinemia with jaundice. He reported fatigue, anorexia, and generalized weakness developing over the previous week. In his medical history, he has major depression treated with fluoxetine, sigmoid volvulus surgery in 2022, and no drug allergy. He denied any known drug allergies, recent history of blood transfusions, or intravenous drug use. A magnetic resonance performed at another facility excluded obstruction of the main biliary tract. He declared a two-week trip to Africa without antimalarial prophylaxis in the previous two months. Diagnosis of PF malaria was made by rapid polymerase chain reaction test.

The patient was transferred to the intensive care unit (ICU) due to worsening general conditions: he was dazed, asthenic, tachypneic (respiratory rate: 26-28, SpO_2_: 91%-92%), hyperpyretic (>38°C), and jaundiced but hemodynamically stable with valid diuresis. SpO_2_, heart rate, invasive blood pressure, body temperature, respiratory rate, and five-lead ECG were continuously monitored. He started high-flow nasal cannulas for oxygen supplementation.

Upon admission, blood chemistry tests showed the following: procalcitonin (PCT), >100 ng/mL (normal value: <0.05 ng/mL); C-reactive protein (CRP), 20 mg/dL (normal value: <1 mg/dL); hemoglobin (Hb), 8 g/dL (normal value in man: 13.8-17.2 g/dL); total bilirubin, 32 mg/dL (normal value: 0.3-1.2 mg/dL); direct bilirubin, 20 mg/dL (normal value: <0.3 mg/dL); pancreatic amylase, 241 IU/L (normal value: 10-150 IU/L); lipase, 253 IU/L (normal value: 10-140 UI/L); and creatinine, 1.6 mg/dL (normal value: 0.7-1.3 mg/dL).

As per our protocol, cultural tests were conducted, including rectal, nasal, and throat swabs for sentinel germs, blood and urine cultures, and tracheal aspirate. Additionally, a microscopic examination of blood smears was performed, revealing a PF parasitemia level of 30%.

Our infectious disease specialist indicated the administration of artesunate 240 mg at times 0 hours (T_0_), 12 hours (T_12_), 24 hours (T_24_), and 48 hours (T_48_).

During artesunate therapy, daily monitoring of the Q-T segment was performed. Further drug therapy included pantoprazole (40 mg/day IV), enoxaparin (6000 IU/day SC), acetylcysteine (20 mg/kg/day IV in two divided doses), methylated gabesate (1 g/day IV), and total parenteral nutrition with adequate caloric intake.

**Figure 1:**
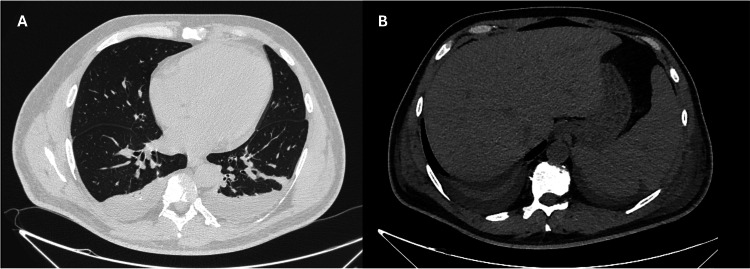
Computed tomography scan (A) The chest view shows bilateral pleural effusion. (B) The abdominal view shows perihepatic effusion, particularly in the subphrenic and paracolic regions. The pancreas is normal, showing no signs of inflammation, edema, or involvement in the pathological process.

The computed tomography total body scan without and with iodinated contrast medium showed moderate bilateral pleural and perihepatic effusions (Figure 1).

On the second day, peripheral blood thick and thin smears revealed a parasitemia of approximately 15%. On the third day, due to persistently high direct and indirect bilirubinemia, inflammation indices, and persistent altered neurological status, EHA with a CytoSorb® filter was started for 24 hours under heparin continuous infusion (4 IU/Kg/hour), monitoring the plasma antithrombin III (AT III) (target: 70%) and activated clotting time (ACT) (target: 190-220 seconds) values every six hours.

**Figure 2:**
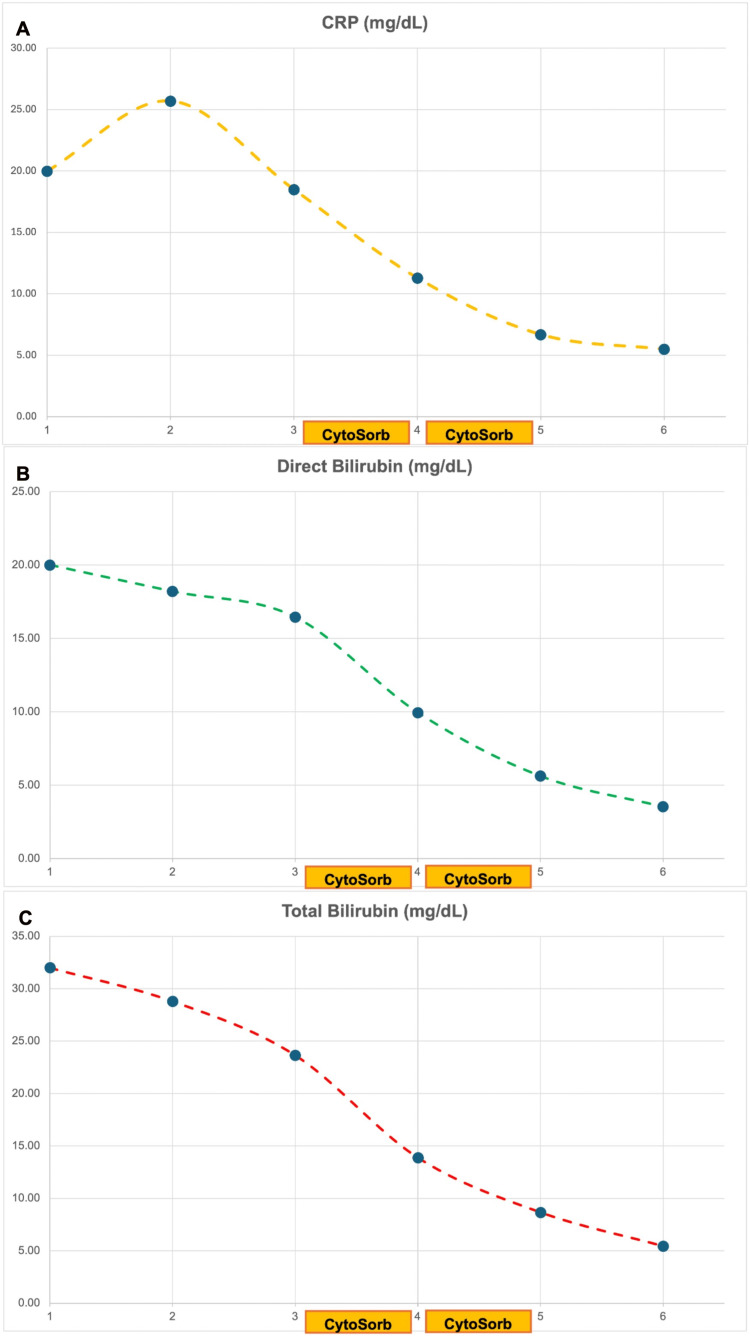
Laboratory parameter trends The figure shows the trends for CRP (A), direct bilirubin (B), and total bilirubin (C) over a six-day period. The application of CytoSorb® on the third to fifth days shows a marked decrease in total and direct bilirubin, as well as CRP, indicating the effectiveness of hemoadsorption technology in improving patient biochemical markers in severe malaria-induced liver dysfunction. CRP: C-reactive protein

After the first EHA cycle, laboratory tests showed a drastic reduction in total bilirubin (13.9 versus 23.6 mg/dL), direct bilirubin (9.9 versus 16.4 mg/dL), CRP (11.3 versus 18.5 mg/dL), and PCT (25 versus 83 ng/mL) (Figure 2). Furthermore, a significant reduction of jaundice and respiratory distress and normalization of the neurological status was evident. However, a blood transfusion was performed due to anemia (Hb: 7.6 g/dL, HCT: 30%). Diuresis was valid during and after the EHA treatment.

A second blood smear analysis showed the presence of very few parasites. On the fourth day, a second 24-hour EHA treatment was performed, and laboratory tests and clinical conditions improved further.

On the fifth day, therapy with atovaquone/proguanil (250 mg/100 mg, one tablet/day) was started for three days. Table 1 presents the trends of key laboratory parameters over the first six days from ICU admission.

**Table 1:** Laboratory trends over the first six days from ICU admission Antimalarial therapy started on day 1 and EHA with CytoSorb® on days 3 and 4. ICU: intensive care unit, CRP: C-reactive protein, n.v.: normal value

Parameter	Day 1	Day 2	Day 3	Day 4	Day 5	Day 6
CRP (n.v.: <1 mg/dL)	20	25.7	18.5	11.3	6.7	5.5
Direct bilirubin (n.v.: <0.3 mg/dL)	20	18.2	16.4	9.9	5.6	3.5
Total bilirubin (n.v.: 0.3-1.2 mg/dL)	32	28.8	23.6	13.9	8.7	5.4

On the eighth day, the patient's clinical condition improved, and he was transferred to the ordinary infectious diseases ward. During the ICU length of stay, the patient never required hemodynamic support and non-invasive or invasive mechanical ventilation. No further complications occurred during the length of stay, and 14 days after ICU discharge, the patient returned home.

## Discussion

**Figure 3:**
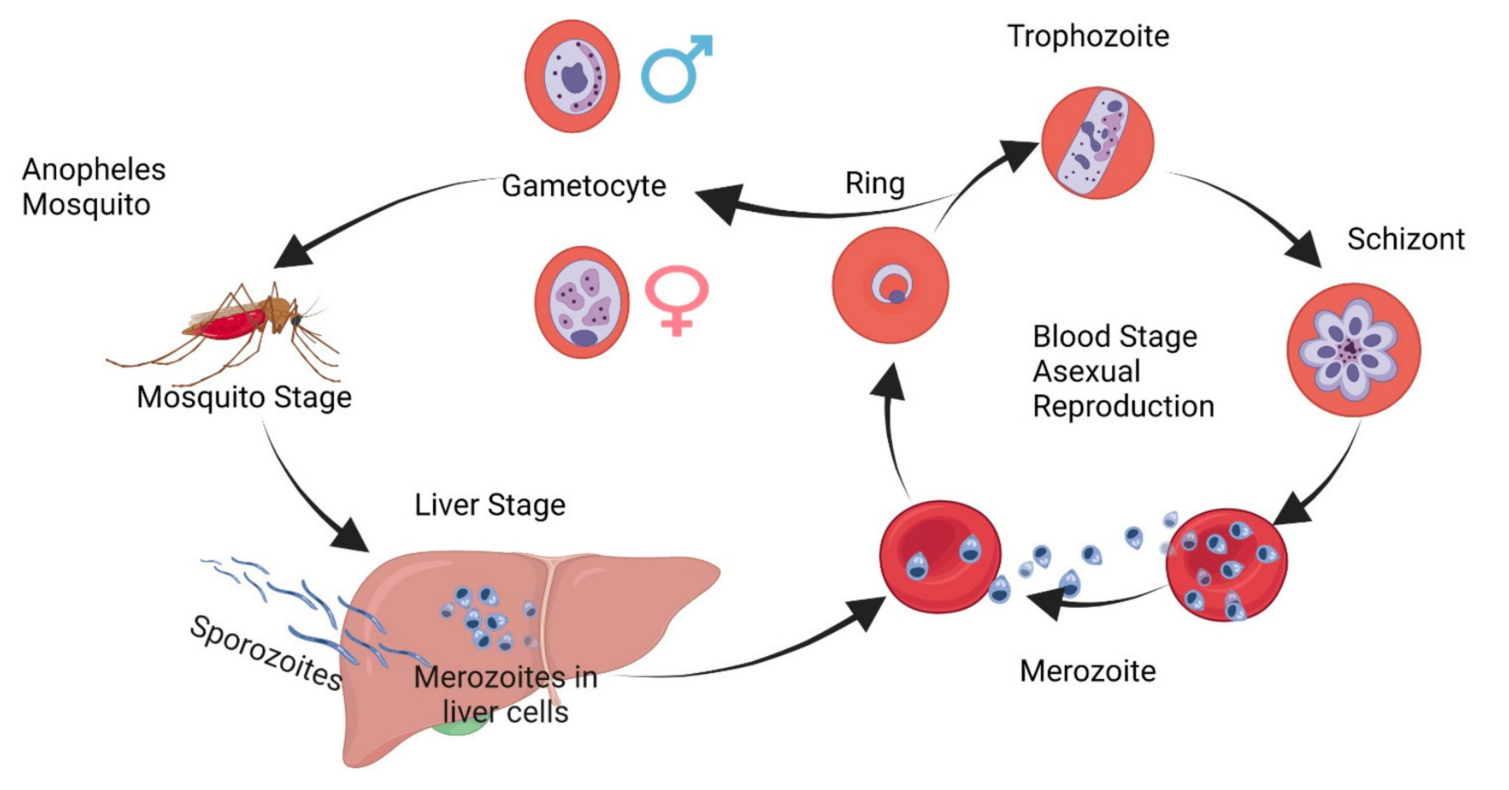
Life cycle of Plasmodium parasite The figure shows the complex life cycle of the *Plasmodium* parasite responsible for malaria. The cycle starts with bites of infected female *Anopheles* mosquitoes. Sporozoites infect hepatocytes, and after developing in the liver, merozoites are released and infect red blood cells, undergoing several stages of development before re-entering the mosquito upon blood meal. The cycle completes with the sexual reproduction stages in the mosquito's midgut, leading to the production of new sporozoites. The image is published under Creative Commons Attribution 4.0 International License (Simoiu et al. Cells 2023, doi.org/10.3390/cells12172156).

Severe malaria, commonly caused by PF, manifests when parasitemia levels are high, leading to complications such as cerebral malaria (altered consciousness or seizures), severe anemia, metabolic acidosis, hypoglycemia, acute kidney injury, respiratory distress, and severe liver dysfunction [7]. Hepatocellular dysfunction, with hyperbilirubinemia and an increase in transaminases up to fulminant liver failure, is described in PF malaria (Figure 3).

Hyperbilirubinemia can cause brain oxidative damage, particularly in the cerebral cortex, basal ganglia, and cerebellum [8]. The term "malarial hepatitis" is used to describe the jaundice caused by malaria, but its pathogenesis is not fully understood. The cytoadhesion of parasite-invaded red blood cells to the vascular and sinusoidal endothelium, leading to "stagnant anoxemia," could be the cause of liver damage caused by PF malaria [9].

**Figure 4:**
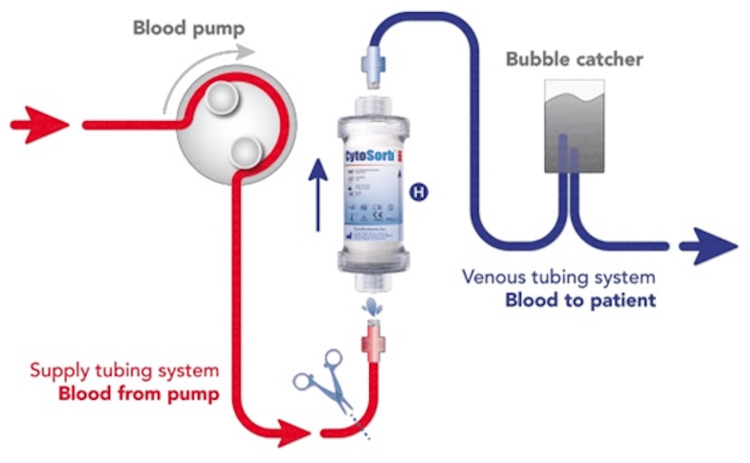
Setup of the CytoSorb® hemoadsorption system The figure shows the setup of the CytoSorb® hemoadsorption system used for removing toxins from the blood. Blood is drawn from the patient using a pump and passed through the CytoSorb® filter where toxins are adsorbed. The cleaned blood then passes through a bubble catcher to ensure safety before being returned to the patient. The image is published under Creative Commons Attribution 4.0 International License (Mair et al. Journal of Cardiothoracic Surgery 2024, doi.org/10.1186/s13019-024-02772-1).

EHA with the CytoSorb® filter (Figure 4) can be used successfully for the plasma removal of pro-inflammatory substances and bilirubin [10].

In sepsis, a dysregulated host response against the pathogens causes a "cytokine storm" and an uncontrolled release of mediators with systemic inflammatory response syndrome and organ dysfunction. Intensivists can use EHA techniques, with different hemofilters and adsorption membranes, to decrease circulating levels of endotoxins and pro-inflammatory cytokines, mitigating their detrimental systemic effects [11-13]. Although the application of CytoSorb® in malaria cases is less documented, its utility in managing severe malaria-related complications, such as hyperbilirubinemia, liver dysfunction, and inflammatory response dysregulation, is emerging [5,6]. Severe malaria often mimics septic conditions, making EHA with CytoSorb® a potential adjunctive treatment to address the systemic inflammatory response and multiorgan failure seen in these patients.

One of the most important features of hemofilters is their sieve cutoff, which is the smallest molecular weight of a solute that a membrane can retain [14,15]. CytoSorb® is a special extracorporeal hemofilter that uses highly porous pyrrolidone-coated polystyrene-divinyl-benzene polymer beads to remove low molecular weight and middle molecular weight (10-55 kDa) cytokines. It has a very high removal rate (>95%) of pro- and anti-inflammatory cytokines, including IL-6, IL-10, IFN-γ, and TNF-α, due to its large surface of adsorption and pore capture. Therefore, it is particularly indicated in clinical settings of elevated cytokinemia. Furthermore, the device consists of a suspended column of highly porous resin covered with a biocompatible coating, capable of removing over 90% of the bilirubin (0.7 kDa) that passes through it [11]. However, CytoSorb® does not remove endotoxins, as opposed to Toraymyxin™ (Toray Industries Ltd., Tokyo, Japan) [16,17]. The Toraymyxin™ hemofilter consists of polystyrene fibers with immobilized polymyxin B, a polycationic antibiotic that binds to the electronegative lipid A portion of the endotoxin, neutralizing its toxicity [18]. In addition to these peculiar characteristics, the two filters also differ in their application times: the CytoSorb® session lasts 24 hours, while the Toraymyxin™ session lasts 120 minutes. Clinicians can perform a second cycle of both after 24 hours from the first treatment.

With respect to the different removal and absorptive features of the various hemofilters, Malard et al. published an interesting study comparing three single-use blood purification filters (Oxiris™ (Baxter International Inc., Deerfield, IL), CytoSorb®, and Toraymyxin™) in terms of removing sepsis-associated mediators and endotoxins [16]. They performed, in vitro, the hemoperfusion of heparinized human plasma from healthy volunteers, which was preincubated with pathological quantities of inflammatory mediators, and analyzed the removal rates of the various cytokines and endotoxin after the hemofiltration. The three filters showed different adsorption and clearance capacities. Oxiris™ was the only hemofilter tested that exhibited the broadest adsorption capacity, with both cytokine and endotoxin (i.e., lipopolysaccharide) removal, with similar endotoxin removal to Toraymyxin™ and similar cytokine clearance to CytoSorb®. On the other hand, Toraymyxin™ showed only lipopolysaccharide adsorption ability, with no cytokine clearance, and CytoSorb® showed only cytokine removal, with no lipopolysaccharide clearance.

This study further endorses our choice to use the CytoSorb® hemofilter because our patient showed an infectious clinical picture where hyperbilirubinemia represented an extremely dangerous condition without acute kidney injury and with normal endotoxin activity assay. A single 24-hour treatment enabled us to significantly reduce the plasma concentration of total and direct bilirubin. We decided on a second cycle to stabilize the result obtained.

## Conclusions

In conclusion, the use of the EHA is safe and effective in achieving a favorable outcome for patients with PF malaria waiting for the effect of antimalarial therapy. The bilirubin removal by CytoSorb® hemofilter allowed us to create a "bridge" for specific therapy, improve symptoms, and prevent organ damage. This case supports further exploration of the use of EHA with CytoSorb® in managing severe malaria cases. Specific areas of research could include evaluating its impact on inflammatory markers, bilirubin clearance, and organ function recovery in patients with malaria-induced multiorgan dysfunction. Additionally, studies should investigate optimal timing, duration of therapy, and patient selection criteria to maximize the benefits.
